# Decolonising global health research: Shifting power for transformative change

**DOI:** 10.1371/journal.pgph.0003141

**Published:** 2024-04-24

**Authors:** Ramya Kumar, Rajat Khosla, David McCoy

**Affiliations:** 1 United Nations University-International Institute for Global Health, Kuala Lumpur, Malaysia; 2 Department of Community and Family Medicine, Faculty of Medicine, University of Jaffna, Jaffna, Sri Lanka; McGill University, CANADA

## Abstract

Recent debates on decolonizing global health have spurred interest in addressing the power asymmetries and knowledge hierarchies that sustain colonial ideas and relationships in global health research. This paper applies three intersecting dimensions of colonialism (colonialism *within* global health; colonisation *of* global health; and colonialism *through* global health) to develop a broader and more structural understanding of the policies and actions needed to decolonise global health research. It argues that existing guidelines and checklists designed to make global health research more equitable do not adequately address the underlying power asymmetries and biases that prevail across the global health research ecosystem. Beyond encouraging fairer partnerships within individual research projects, this paper calls for more emphasis on shifting the balance of decision-making power, redistributing resources, and holding research funders and other power-holders accountable to the places and peoples involved in and impacted by global health research.

## 1. Introduction

Inequity within international research partnerships has troubled the field of global health for decades. In particular, power asymmetries between actors from wealthier and historically-privileged countries and their counterparts in the Global South (GS) have led to paternalistic ways of working, unequal sharing of resources, skewed distribution of benefits, and limited commitments to capacity strengthening [1]. Recent debates on decolonizing global health have brought renewed attention to addressing these problems in global health research. In addition to highlighting equity concerns, these discussions draw attention to the epistemic injustice and “white saviour” mentalities that underpin research collaborations [2–8].

Recognising that power asymmetries in global health are produced by both historical and current exploitation and resource extraction, our approach to decolonizing global health involves three intersecting dimensions: 1) colonialism *within* global health; 2) colonisation *of* global health; and 3) colonialism *through* global health [9]. The first dimension speaks to power differentials and resource disparities between different actors *within* the field of global health. The second deals with the dominance of certain powerful actors and vested interests over the overall complex of global health structures, systems, policies and practices. The third dimension refers to exploitative and extractive practices that occur through the health sector [9].

**Fig 1:**
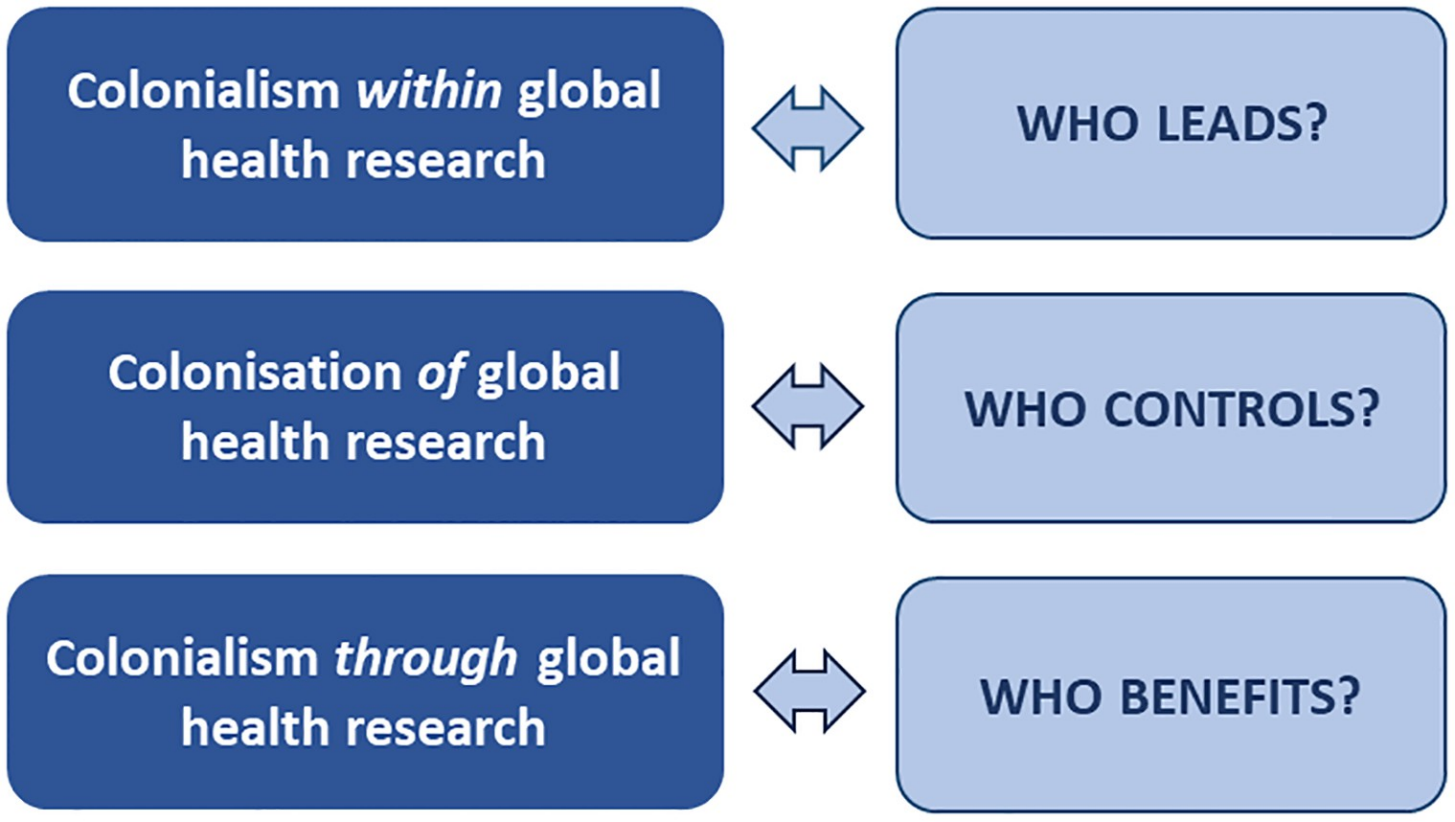
Intersecting dimensions of colonialism in global health research.

This paper uses this framework of three dimensions to arrive at a broader understanding of the scope of policies and actions needed to decolonise global health research. We begin by briefly outlining persisting inequities *within* research partnerships- already addressed by a large body of literature. Next, we draw attention to issues that are underexplored, specifically who controls the agenda of global health research (i.e., colonisation *of* global health research), and who benefits from such research (i.e., colonialism *through* global health research) (Fig 1). We then present a brief review of recent guidelines and checklists that seek to decolonize global health research and/or centre the needs and aspirations of the GS in research, revealing an emphasis on addressing inequity *within* research partnerships. We end by recommending policies and actions that would decolonize the field of global health research in an effective and comprehensive manner.

This paper employs the terminology “Global North” (GN) and “Global South” (GS) to reflect asymmetries in power and access to resources between not just countries but also population groups. This terminology only partly corresponds to the classification of countries according to per capita gross national income, i.e., low-income, middle-income, and high-income countries (LIC, MIC, and HIC) [10]. However, where we quote from sources that explicitly refer to LICs, MICs or HICs, these terms are retained. We borrow from Garcia-Basteiro and Abimbola [11] to define global health research as research that seeks to address health inequity within and across countries, aiming to improve health in what they call “low-resource settings” described as regions weighed down by financial constraints, suboptimal service delivery, underdeveloped physical and knowledge infrastructure, historical, political and sociocultural contexts/specificities, and geographical, environmental and human resource limitations.

## 2. Colonialism *within* global health research: Who leads?

According to the World Health Organization (WHO), in 2020, of the USD 37 billion spent worldwide on ‘biomedical research’, 98.7% went to HICs [12]. Perhaps more reflective of the global health research landscape, in 2021, 82% of the Bill and Melinda Gates Foundation’s (BMGF) grant funding went to HIC recipients [13]. This unequal distribution of funding is striking when one considers that much global health research is carried out in GS settings.

The inequitable global health research funding patterns reflect not only the wider socio-economic disparity between GN and GS, but also the biases within the global health research system. For example, grant calls, either explicitly or through eligibility criteria or capacity requirements, favour GN-based institutions [14] with research funding agencies of key donor countries often requiring principal investigators (PI) to be based in their country or compel PIs from the GS to partner with a researcher based in the donor country [15, 16]. Eligibility criteria based on geographic location and experience may further restrict applications from GS-based researchers [17]. GN-based researchers are also better able to navigate the funding terrain with their training, networks and resources [18, 19].

Although most global health funding agencies require GN-based researchers to “collaborate” with local “partners,” the terms of collaboration are usually set by the former who typically conceptualise the research before inviting others onboard [20]. This gives GS-based researchers limited influence over the research, despite their expertise and familiarity with the context [7, 21, 22], thus supporting what has been called “parachute” research, where GN-based collaborators fly in for weeks at a time for onsite “supervision” [23, 24]. As grant cycles are usually short, the urgency to meet deadlines results in lopsided decision-making, hasty administrative approvals and, at times, the undermining of local administrative and ethics procedures [8].

Much grant funding goes towards the salaries of GN-based researchers with substantially less dedicated to research systems and capacity strengthening in GS settings [2, 14]. This lack of long-term commitment to the development of GS-based institutions sustains the status quo [25]. Meanwhile, extant capacity strengthening initiatives are often uni-directional and paternalistic, involving assumptions about what competencies GS collaborators may lack [26].

Inequity is further reinforced by authorship patterns that are biased towards GN-based researchers [27, 28]. Authorship guidelines of prominent journals systematically exclude non-native English writers [29] by giving weight to written contributions over field work [30]. Representation at conferences and symposia is similarly unequal, although research collaborations do enable participation for some GS-based researchers. Even so, visa and other barriers challenge researchers from travelling to meeting destinations [31].

## 3. Colonisation *of* global health research: Who controls?

Global health funding agencies wield significant power in defining global health problems and the approaches taken to addressing them [7, 32]. Under the current system, researchers based at universities and other research institutions respond to grant calls, crafting their research to fit with the agendas and ideologies of global health funders rather than vice versa [33].

Extreme wealth concentration under neoliberal globalization and the rise of ‘philanthrocapitalism’ by which global health problems are framed as market opportunities, has seen a shift from public to private financing in global health [34, 35]. However, private actors have interests and priorities that may be at odds with the public interest or with achieving equity in health. For instance, the shift from publicly-funded to industry-funded research has distorted scientific evidence on infant formula with detrimental effects on infant and child health [36]. Moreover, the funding decisions of corporations and foundations are ultimately approved by a handful of largely GN-based board members, who are not subjected to any independent mechanisms of accountability for their funding decisions or their impact on people affected by these decisions [32, 37]. Although some funders have recently instituted measures to address diversity within their leadership [13, 38], such change will not be transformative without redistributing power and resources, and genuine efforts to improve accountability [39].

Research funders favour specific thematic areas, not always based on the health problems prevailing in specific GS settings [19, 40]. They tend to promote technology-based solutions and favour innovation and entrepreneurship in projects that yield quick and quantifiable results [41]. The preference for short-term impact over longer-term improvements in health results in grant proposals that centre “magic bullets” (e.g., vaccines, medicines, bed nets, mobile apps) rather than systems building, local capacity strengthening and unblocking the social and political barriers to the scale up of proven and more sustainable alternatives [42, 43].

Academic programmes in global health continue to be characterised by what has been called a “white saviour complex” or a depoliticized, patronizing and charity-based approach shaped, in part, by a wider aid industry [44, 45]. Global health curricula remain largely disconnected from the many realities and locales of the GS, both in geography and lived experience. Dominant Eurocentric epistemologies, which are embraced and propagated by powerful global health institutions, are usually given primacy in research training, even as heterodox methodologies that interrogate power and inequality are marginalized [45, 46].

The inability of countries of the GS to weigh in on the global health research agenda and define their own priorities is perpetuated by their minimal contributions to research funding [25, 47, 48]. While domestic investment is critical to shift the balance of power, debt-ridden governments of lower-income countries may have limited leeway with their health and R&D budgets owing to fiscal constraints [19]. For these countries, HIC-driven global health research collaborations may present a welcome source of foreign currency. Too often however, external funding for health research takes place with little coordination among granting agencies [49, 50], facilitating duplication, and making impact assessment difficult.

## 4. Colonialism *through* global health research: Who benefits?

The asymmetric global health research funding structure also gives powerful states and private actors opportunities to craft research in the GS in ways that they benefit from financially or economically. These benefits are primarily driven by the commercialization of research and publishing, supported by imperatives to expand markets, unfair intellectual property rights (IPR) regimes, and predatory academic journals.

Arguably, the biggest profits are made by commercial entities that hold patents for global health technologies often tested through research carried out in GS settings. Such research aids market expansion for medicines, vaccines, diagnostic tests, mobile devices, etc. benefitting big pharma, biotechnology, and big tech companies, while doing little to strengthen public health infrastructure and services or reduce dependency [19, 41]. Indeed, some private foundations are routing a growing proportion of their tax-subsidised grants to private for-profit organisations, in both GN and GS settings [37, 51].

Current IPR regimes which provide private companies with extensive monopoly rights over new and modified technologies despite much basic research being funded publicly is one aspect of an R&D ecosystem biased in favour of private financial interests at the expense of public health. This was seen with the billions of dollars of private profits generated from COVID-19 vaccines despite vast amounts of public and charitable funds that went into their development [52].

The unequal benefits accrued through authorship in global health journals have been widely studied [27, 28] but less is known about their commercial dimensions. The revenue of academic publishers is estimated to be about USD 19 billion annually, where about half the market share is controlled by five transnational companies, with Elsevier alone accounting for 16% of the market share, with profit margins in the order of 40 per cent [53]. These corporations are all headquartered in the GN and maximise profits through article processing charges (APCs), subscriptions, and the uncompensated labour of authors and peer-reviewers. Ever-increasing APCs are required to publish ‘open access’ in prestigious journals, implemented in the name of equity, but barring most GS-based researchers through stringent waiver criteria [54]. Global spending on APCs alone is estimated to exceed USD 2 billion annually [55]. Academic journals are, in turn, linked to bibliometric platforms that track the ‘impact’ of research communications, which feed into commercialised university ranking systems [56]. With research funding and citations in ‘high-impact’ journals being key elements of performance indices, the top-twenty universities, as ranked by Academic Ranking of World Universities and Time Higher Education, are all located in the GN [57].

The current system of global health education supports extraction of wealth and other resources from GS to GN. A recent analysis of masters in global health degrees revealed that 95% of them are based in HICs, costing on average USD 37,732 in tuition [58]. Given the location and cost of global health postgraduate programmes, their graduates, including those from GS settings, are likely to be drawn to work with global or GN-based institutions both to repay the debt incurred and because of the lack of well-remunerated positions back home [58]. Ultimately, career trajectories in global health are skewed towards the GN and not “low-resource settings” where global health work and resources are much needed [23].

In sum, whether in terms of leadership, control or benefits, GN-based actors and institutions are privileged within the broader global health research ecosystem, often to the detriment of researchers, institutions and ‘beneficiaries’ in GS settings. It appears that global health research supports a renewed form of extractivism, where resources in the GS, including funding, knowledge and researchers, are drawn to the GN. In the next section, we examine whether and to what extent recent guidelines on decolonising global health research address the three intersecting dimensions of colonialism in global health research.

## 5. Recent guidelines that aim to decolonise global health research

We searched the literature for tools that either explicitly or in their framing seek to decolonise global health research and/or centre the needs and aspirations of the GS in research. As searches on PubMed and Scopus [(“decol*” OR “colonial*”) AND “global health” AND “research” AND (manual OR guideline OR checklist)] yielded less than 10 publications, we also searched Google Scholar, Google, and pursued reference lists of identified publications. Criteria for inclusion were: addressing equity in global health research with reference to colonialism or explicit attention to making research fairer for peoples and institutions in the GS; including a set of standards or guidelines; targeting researchers, research institutions or funders; published within the five-year period of 2019 to 2023. We identified eight tools that fit our criteria as described below.

Hodson et al. [40] offer a set of “practical measures” for global health researchers, underpinned by four principles: “1) seek locally derived and relevant solutions to global health issues, 2) create paired collaborations between HIC and LMIC institutions at all levels of training, 3) provide funding for both HIC and LMIC team members, [and] 4) assign clear roles and responsibilities to value, leverage, and share the strengths of all team members.” This guideline addresses specific challenges experienced in GS settings by advocating for: educating all team members on global health history; early engagement of GN-based researchers with local administrations; capacity strengthening to support independent research in GS settings; protected research time for all team members; preventing GS-based researchers being drawn away from regular work; and ensuring knowledge translation to local communities, among other measures. Despite the commitment to long-term capacity strengthening, the guideline focuses primarily on research processes within partnerships.

Kumar et al. [26] propose a set of individual and institutional level actions to advance equity in global health research. Those at the individual level include questioning “notions of absolute scientific objectivity” (p.146), adopting a decolonial approach towards global health concepts and implicit hierarchies, cultivating respect and humility, promoting fairness at all levels (including at the level of global health leadership), and going beyond ‘equality’ to recognize ‘equity’ within collaborations. At the institutional level, they support decentring the GN in global health efforts (including the location of centres of knowledge), promoting solidarity, investing in researchers from LMICs, bi-directional capacity strengthening, evaluating partnerships by “measures of fairness” and “ethical and culturally responsive engagement,” and correcting “colonising and unethical practices” (p.146). While some of these actions aim to rectify power asymmetries well beyond research partnerships, they do not include specific guidance on implementation.

Embracing a feminist decolonial approach, Singh et al. [59] offer a guideline for researchers working in situations of forced displacement that centres participant agency, voice, and experience; it aims to address power hierarchies through a set of recommendations targeting various stages of research. The guideline demands: consideration to “political, social, economic, and historical contexts and power hierarchies of the research setting” (p.561); involving marginalised groups in the research design; reflecting on how coloniality and gendered power relations may be reinforced during data collection; an intersectional analysis of gendered power relations; collaboration in analysis and knowledge dissemination; and using research to “challenge unjust systems and policies and deliver gender transformative and equitable programmes” (p.561). Although the guideline aims to reconfigure power within individual research projects, it offers no direction on how to redistribute power.

Rashid [8] offers guidance for researchers in LICs to “[navigate] the violent process of decolonisation in global health research.” The guideline includes a list of dos and don’ts to help researchers in LICs contend with power asymmetries in international research collaborations. They recommend carefully reviewing agreements, clarifying systems of reporting and accountability, insisting on inclusion in communications with funders, meticulous documentation, boosting one’s profile, expanding networks, and building solidarity. However, this guideline focuses on change at the individual level on the part of researchers in GS settings rather than systemic change.

The TRUST Code–“A Global Code of Conduct for Equitable Research Partnerships” is based on the core values of fairness, respect, care, and honesty [60]. Compiled by a team with wide representation from the GS, the TRUST Code consists of 23 articles. Apart from conventional ethical standards, the tool emphasises: bona fide involvement of local communities in research, fairness in the transfer and ownership of data and biological materials, and fair compensation of local collaborators. It emphasises cultural acceptability, community assent, respect for local ethics review and giving consideration to the impact of research on local human resources, animal welfare, and the environment. It calls for clarity on roles, responsibilities, capacity strengthening, transparency, and integrity of the research process. Although broadly framed around justice for communities and researchers in the GS, the tool primarily concentrates on making individual research partnerships more equitable.

The Research for Health Justice Framework proposed by Pratt and colleagues [61] offers two sets of guidelines, one for health researchers and another for granting agencies. Developed through an iterative process and fine-tuned through case studies in GS settings, the guidelines emphasize equity, justice, and inclusion, with accompanying explanations on implementation. The guideline for researchers addresses: selection of the research population and research problem, research capacity development, delivery of ancillary care, and knowledge translation practices. With respect to granting agencies, it asks that they prioritise the health concerns of the worst-off, promote ownership of the research agenda by LMIC researchers and support projects that seek to advance equity within healthcare systems, atop measures to support equitable research practices. While this framework is comprehensive in scope, the guidelines are still largely limited to the research process and do not explicitly seek to transform the global health granting system and the power asymmetries within it.

Focusing specifically on global health research funding, Charani et al. [19] outline eight areas of action for funders: 1) developing situational awareness, including an understanding of institutional dynamics and who benefits from grants; 2) formulating a mission statement that pledges equity in research; 3) equitable allocation of funds to cover differential needs of HIC- and LMIC-based researchers; 4) funding structures that encourage local ownership and leadership; 5) bi-directional capacity strengthening that enables all partners to engage with funders; 6) diversity and inclusion across the grant cycle, including in design, knowledge dissemination, access to training etc.; 7) knowledge generation, including methodologies, frameworks, tools and clarity on data ownership; and 8) reflection and feedback involving HIC and LMIC researchers on equal terms. Encouraging funders to include specific requirements for grant recipients to comply with participatory approaches and fair sharing of resources and benefits, the guideline also speaks to what should be funded, who should be funded and how. Moreover, among its recommendations—albeit with no details provided—are “a transparent process for tracking the progress of funding” and “a code of ethics for global health funders”.

The Global Health Decolonisation Movement Africa [17], self-described as a collective of African citizens, has published a guideline called, *Pragmatic Approaches to Decolonising Global Health in Africa*. What is unique about this guideline is that it addresses multiple “stakeholders” in HICs, including individual practitioners, funding agencies, academic and training institutions, scientific publishers, and event conveners and organisers, among others. The guideline broadly seeks to address racism against Africans within global health, and promotes African leadership and self-determination. The section for funders calls for diversifying grant review panels, rejecting “parachute” proposals, and removing requirements for researchers based in Africa to collaborate with HIC-based institutions. For academic and training institutions, the guideline recommends diversifying leadership and recruitment practices, and addressing coloniality in global health curricula. And for scientific journals, it demands diversifying authorship and peer-review panels. While this guideline emphasises diversity, equity, and inclusion, it remains constrained by the limitations of the current system of global health research funding.

In sum, there is considerable variation in guidance on improving equity in research partnerships and decolonising global health research. All reviewed sources strive to make the research process fairer and rectify power asymmetries through diversity, equity and inclusion measures, but only some engage with historical imbalances in power, interrogate dominant knowledge paradigms, centre the concerns of marginalized groups, and create space for self-determination. The guidelines for funders go beyond research partnerships to address who and what is funded. However, for the most part, these guidelines neglect the wider contextual factors that shape agenda-setting in global health research, as well as the actors and institutions that control and benefit from them.

## 6. Shifting the balance of power in global health research: Going forward

**Fig 2:**
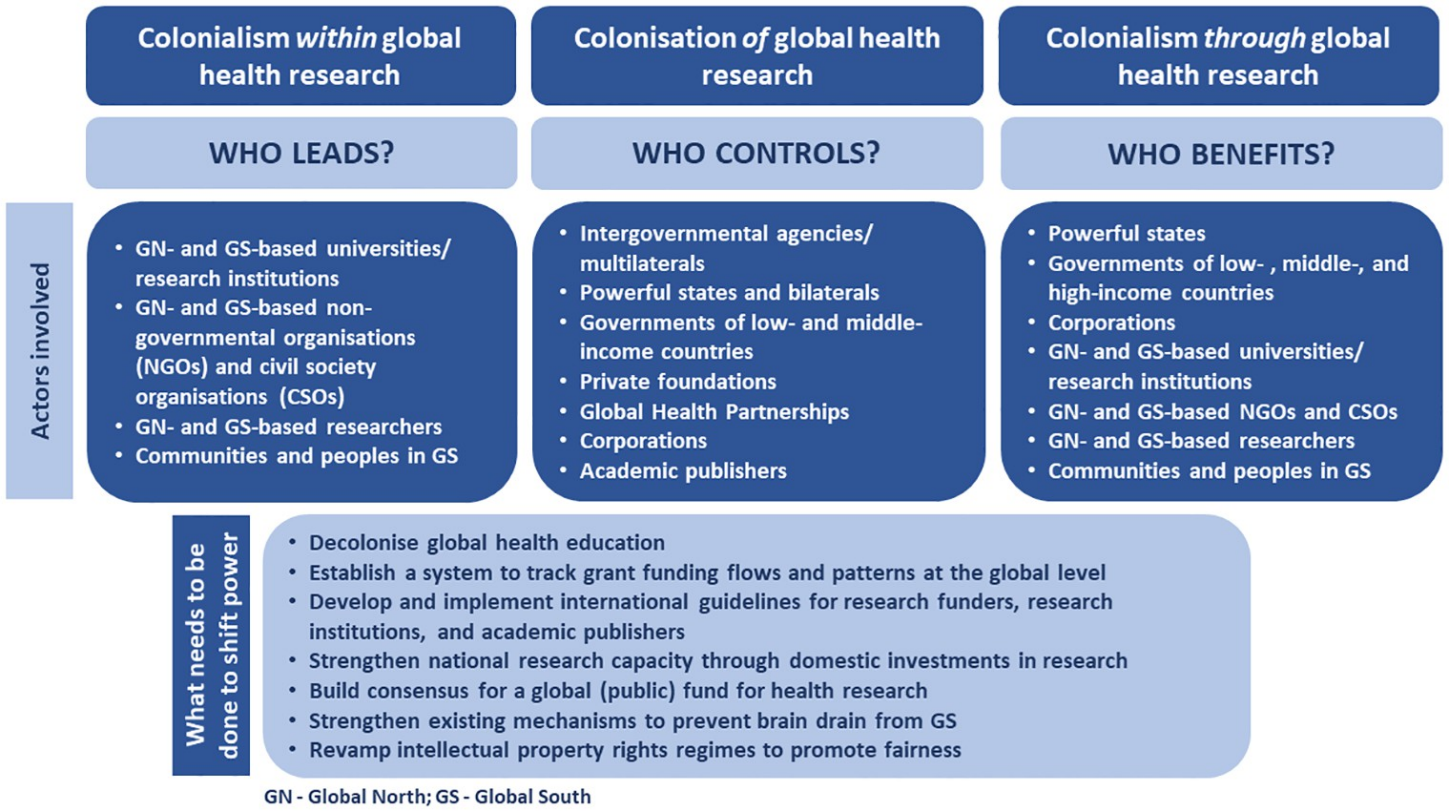
Actors and action areas to be targeted to shift the balance of power.

In this section, we draw on the three intersecting dimensions of colonialism in global health research to present seven action areas that we call for to mitigate inequitable, exploitative and extractive arrangements in global health research (Fig 2).

First, and most fundamentally, we call for a critical examination of the epistemological and ideological underpinnings of global health research. While current debates engage to some extent with the marginalization of indigenous perspectives, few question the dominance of positivist approaches and the biomedical paradigm. Guided by a biased hierarchy of evidence that favours quantitative assessments, global health research remains over-occupied with testing the efficacy of discrete, downstream and often clinical technologies and interventions, taking attention away from the social and structural determinants of health, which are more challenging to measure [41, 43, 62]. Shaped by neoliberal ideology, understandings of health and healthcare have evolved from collectivist to individualist interpretations, giving way to economistic evaluations based on assumptions that resource constraints in low-income settings are inevitable [63]. Global health education could challenge dominant paradigms and mainstream approaches that advance social justice and equity in health [64].

Second, we need a better and more detailed analysis of the overall pattern and performance of research funding: where it comes from, where it goes, how it is spent, and its impact. A few of the guidelines reviewed earlier do address the global health research funding system. For instance, Charani et al. [19] recommend that funding agencies self-monitor whom they fund and also call for a code of ethics for funders, while the Global Health Decolonisation Movement Africa [17] asks funders to remove requirements for researchers based in Africa to collaborate with GN-based institutions. Even so, these measures remain couched within the current structure and system of grant funding that lacks transparency and leaves power concentrated in the hands of largely GN-based donors. The problematic norm of donors funding favoured research areas over those that are identified locally remains largely unchallenged. At the very least, information should be available by funder, recipient, research area, and research setting, possibly through a centralized system that requires funders to provide information on their funding practices. Auditing such data should enable analysis of not only where research funding comes from and who receives it but also its impact.

Third, efforts to address power asymmetries in global health research must compel reform at the highest levels of global governance. By virtue of their funding contributions, powerful states, their bilateral agencies, private foundations, and corporate actors, among others, shape the global health research agenda. Bilateral agencies tend to push foreign policy and other domestic interests [65, 66], while corporate actors are driven by profit, and many private foundations by the creed that the private sector can more effectively tackle intractable global health problems [67]. Bilateral and multilateral agencies should be held accountable for what they fund with taxpayer contributions, while private funders—who are primarily accountable to their boards—must be appropriately regulated and prevented from having undue influence on the shaping of research priorities [68, 69].

A comprehensive guideline for research funders that promotes fairer distribution of resources and improved accountability is needed. Such a guideline could incorporate the measures proposed by Charani et al. [19], Pratt et al. [61] and the Global Health Decolonisation Movement Africa [17]. An international agreement, akin to the Declaration of Helsinki [70]—the World Medical Association’s ethical principles for medical research—could encourage and eventually normalise funding of equity-oriented research and local ownership. Decision-making on funding priorities must be shared with the GS, not just with governments but also with researchers, institutions, and the beneficiaries of research [26].

Fourth, national research systems should be supported and strengthened with in-built mechanisms of accountability. While there are calls for LMIC governments to invest more in R&D [25], the onus for change cannot be placed on these countries alone. Rather donors must also commit to investing in local research infrastructure, human resources, and higher education systems, all key to building research capacities. Meanwhile, government allocations for health research in GS settings should be guided by appropriate needs assessments and strategic plans to strengthen national research capacity [71] as once encouraged by the Commission on Health Research for Development (COHRED), an independent global initiative that supported research for heath and development in LMICs [72]. Systemic investments in research capacity strengthening with long-term budget commitments and harmonised mechanisms should be established [73] to replace the current piecemeal manner in which health research is conducted, often subject to the whims of external funders. Bi-directional scholarships for postgraduate training in research, with service requirements in GS settings, could target specific human resource gaps. Fifth, a global fund for research [74], guided by a multilateral framework that pools donor funds and channels them based on national health priorities may help to harmonise external funding, avoid duplication, and enable greater transparency and accountability.

Sixth, given acute human resource constraints in many GS countries, brain drain must be stemmed. The WHO Global Code of Practice on the International Recruitment of Health Personnel [75] provides a multilateral framework but fails to hold the GN to account for their unethical recruitment practices. Instead, the Code focuses on the rights of migrating health workers and places the onus on ‘developing countries’ to retain them. It does not recognize the vast amounts of (often public) resources invested in health worker training in GS settings, nor does it recommend compensation to source countries for this training. Academic global health programmes should re-orient their curricula [76] so that the primary career pathways for global health practitioners are viewed to be in GS settings.

Lastly, interventions to promote fairer distribution of benefits should look beyond authorship and academic credit, to address extractivist practices within the research industry that impede access to knowledge and technologies in the GS. The current IPR regime upholds patent protection, allowing big pharma to control product pricing and restrict market entry of generic manufacturers who could drive down the cost of medicines and other health products [77, 78]. IPR regimes need to be revised to enhance fairness in the distribution of the benefits of science rather than support industry benefits and profit over public health.

## Conclusion

In this paper, we applied three intersecting dimensions (colonialism *within* global health; colonisation *of* global health; and colonialism *through* global health) to develop a broader and more structural understanding of the policies and actions needed to decolonise global health research. We highlighted the tendency of existing guidelines that seek to make research partnerships more equitable and less colonial, to target the behaviour of researchers and research institutions within the boundaries of individual research projects. Following such guidance should result in better and more appropriate global health research. However, efforts to decolonise global health research should go beyond addressing equity within research partnerships to reconfiguring power arrangements within the global health research ecosystem. This means re-orienting research along social justice and equity lines, building research capacities in GS settings, and moving away from the existing donor-driven model.

Of critical concern is the prevailing system of research funding that functions with little transparency or downward accountability. Data should be made available to scrutinize and evaluate the funding processes of research funders and the appropriateness and impact of funding patterns and practices. It would be important to examine not just the specific outputs and outcomes of individual grant programmes and research projects, but also the impact of the entire global health research portfolio on the overall functioning of health research systems at global and national levels and, in particular, how research outputs contribute towards advancing health equity. Quick fixes and half-hearted measures would simply not work. Time is now for the global health community to come together and demand a complete overhaul of the competitive global health research funding system, and its replacement or accompaniment with a more strategic and publicly-driven pooling and harmonised allocation of resources aimed at correcting the many deep and structural inequalities across the global health research ecosystem. This would also require fostering equity-oriented research approaches, grounded in local ownership, with systems of accountability built in.
